# The abuse potential of kratom according the 8 factors of the controlled substances act: implications for regulation and research

**DOI:** 10.1007/s00213-017-4813-4

**Published:** 2017-12-23

**Authors:** Jack E. Henningfield, Reginald V. Fant, Daniel W. Wang

**Affiliations:** 1 Research, Health Policy and Abuse Liability, Pinney Associates, 4800 Montgomery Lane, Suite 400, Bethesda, MD 20814 USA; 2 Department of Psychiatry and Behavioral Sciences, The Johns Hopkins University School of Medicine, Baltimore, MD USA

**Keywords:** Kratom, Mitragynine, Abuse potential, Controlled Substances Act, Food and Drug Administration, Analgesic, Opioid, Withdrawal, Dependence, Dietary ingredient

## Abstract

**Rationale:**

Consideration by the US Drug Enforcement Administration and Food and Drug Administration of placing kratom into Schedule I of the Controlled Substances Act (CSA) requires its evaluation of abuse potential in the context of public health.

**Objective:**

The objective of the study is to provide a review of kratom abuse potential and its evaluation according to the 8 factors of the CSA.

**Results:**

Kratom leaves and extracts have been used for centuries in Southeast Asia and elsewhere to manage pain and other disorders and, by mid-twentieth century, to manage opioid withdrawal. Kratom has some opioid effects but low respiratory depression and abuse potential compared to opioids of abuse. This appears due to its non-opioid-derived and resembling molecular structure recently referred to as biased agonists. By the early 2000s, kratom was increasingly used in the US as a natural remedy to improve mood and quality of life and as substitutes for prescription and illicit opioids for managing pain and opioid withdrawal by people seeking abstinence from opioids. There has been no documented threat to public health that would appear to warrant emergency scheduling of the products and placement in Schedule I of the CSA carries risks of creating serious public health problems.

**Conclusions:**

Although kratom appears to have pharmacological properties that support some level of scheduling, if it was an approved drug, placing it into Schedule I, thus banning it, risks creating public health problems that do not presently exist. Furthermore, appropriate regulation by FDA is vital to ensure appropriate and safe use.

**Electronic supplementary material:**

The online version of this article (10.1007/s00213-017-4813-4) contains supplementary material, which is available to authorized users.

## Introduction

Kratom is the most common term in the US for the leaves (whole, chopped, or powdered) and leaf extracts (e.g., tea-like brews, and commercially prepared liquids) of the Mitragyna speciose tree indigenous to Southeast (SE) Asia, Malaysia, and the Philippines. Mytragyna speciose is a member of the Rubiaceae family, which also includes the genus Coffea (Eisenman 2015). Like the coffee tree, kratom contains numerous alkaloids, some of which are active in the central nervous system (CNS) and can produce diverse physiological and behavioral effects which will be discussed in this article (Patay et al. 2016; Raffa et al. 2015). Kratom has been used in SE Asia for centuries in cooking (e.g., to wrap fish, and in stews); however, its most important use has been its oral consumption in the form of tea-like extracts and the chewing of whole leaves in order to enhance mood and well-being; for medicinal and social benefits; and to manage painful demands of physical labor. (Aziz 2014; Swogger et al. 2015).

In mid-twentieth century in SE Asia, kratom was also increasingly used as a substitute for opioids for disorders including diarrhea, cough, pain, and depressed mood states, and to ameliorate opioid withdrawal symptoms in chronic opioid users who were temporarily without access to opioids or who were trying to give up opioids, alcohol, and/or other addictive drugs (Assanangkornchai et al. 2007; Swogger et al. 2015; Grundmann 2017; Singh et al. 2014; Vicknasingam et al. 2010). By the 1990s, perhaps aided by increasing migration of people from SE Asia to Europe and the United States (US), kratom consumption as a natural remedy began to increase outside of SE Asia, facilitated by dissemination of information and marketing on the internet. In 2016, it was estimated that in the US, there were several million consumers purchasing products from more than 10,000 retail outlets with an estimated annual market of 207 million US dollars (Botanical Education Alliance 2016).

Fueled by concerns about the US’s growing opioid crisis as well as the introduction of synthetic psychoactive substances (e.g. K2, Spice, “bath salts”), the US Drug Enforcement Administration listed kratom among its “Drugs of Concern” in 2008 (Drugs Forum 2008). Several states subsequently banned the sale of kratom by placing it in Schedule I along with synthetic cannabinoids and cathinones (American Kratom Association (AKA) and Botanical Education Alliance 2016; AKA 2017; US Drug Enforcement Administration 2017; National Forensic Laboratory Information System 2016b). In 2016, the DEA published its notice of intent to temporarily place mitragynine (MG) and 7-hydroxymitragynine (7-OH-MG) (the primary active alkaloids of kratom) into Schedule I of the Controlled Substances Act (CSA) (US Drug Enforcement Administration 2016; Ingraham 2016). Within weeks, DEA received thousands of comments from the public, a bipartisan response from the US Congress, and legal-regulatory arguments from the AKA by its representatives Hogan Lovells (American Kratom Association and Botanical Education Alliance 2016; Hogan Lovells 2016; Wing 2016). Comments highlighted personal experiences and use in order to abstain from previous dependencies on opioids and to treat a variety of maladies including pain and depression with few risks of side effects and serious adverse events.

In response, the DEA announced less than 2 months after its initial emergency action that it was withdrawing its proposed intent to schedule kratom and would be seeking input from the Food and Drug Administration (FDA), the National Institute on Drug Abuse (NIDA), as well as comments from the public, in order to develop a full abuse potential assessment according to the eight factors of the CSA and develop regulatory recommendations (US Drug Enforcement Administration 2016; Gruley 2016). In general, recommendations for listing a substance or product in one of the schedules of the CSA are ordinarily on such an eight-factor analysis (8-FA). Schedules II though V are for substances that have been approved for medical use with placement in Schedule II (“C-II”) for substances with the highest level of abuse potential, and placement in Schedule V (“C-V”) for those with the lowest level but still determined to warrant control. Schedule I is for any substance that has not been approved for medical use but was determined to warrant control by the US Congress at the time the CSA was developed or was or will be recommended for Control by the DEA, regardless its level of abuse potential. The DEA has a “temporary” or “emergency” scheduling authority that enables it to short cut the usual process “if such action is necessary to avoid an imminent hazard to the public safety.” 21 U.S.C. 811(h)(1) (U.S. Congress 2017) and that analysis was the basis for DEA’s original placement of MG and 7OH in Schedule I of the CSA. Note that if the substance is not approved by the FDA as a medicine (i.e., drug product), then Schedule I is the only option for placement, regardless of its level of abuse potential and regardless of where it would be placed pending the 8-FA (U.S. Congress 2017; Pinney Associates 2016; FDA 2017; Belouin and Henningfield 2017; US Drug Enforcement Administration 2006; Sacco 2014; Spillane and McAllister 2003).

In response to the request for comment by DEA, the AKA commissioned PinneyAssociates to develop an independent 8-FA for submission to DEA, FDA, and NIDA. This article summarizes key elements of the Henningfield and Fant eight factor analysis, with additional research findings that have been published since that analysis was developed, and concludes with recommendations for the future evaluation and regulation of kratom and its primary CNS active alkaloids.

### Factor 1. Actual and relative potential for abuse

Decades of experience in SE Asia leave no question that consumption of kratom can produce effects that people can feel and which are important in their use of kratom. Analysis of Factor 1 provides part of the basis for characterizing the nature and strength of its pharmacological effects and the relevance of this for a scheduling recommendation. Perhaps the most prevailing observations in clinical, scientific, and ethnographic reports from 1930 to 2017 regarding the motivations for kratom consumption are the functional benefits of kratom consumption to enhance, sustain, and even enable occupational work demands. Kratom is/was used for a variety of ailments and disorders that might also be treated with opioids and other substances be they natural medicines or approved drugs; although as of 2017, no kratom product has been filed for approval to the FDA as a drug or listed by the FDA as an approved drug. In the US, it appears that most kratom use is not for abuse related purposes described by the FDA in its abuse potential assessment guidance (FDA 2017, p.4) such as “euphoria, hallucinations and other perceptual distortions, alterations in cognition, and changes in mood”. As discussed in Section 2.5, although a nationally representative survey of the reasons for kratom use had not been done at the time of this writing, four independent surveys found that the vast majority of the respondents reported using kratom to improve health and well-being. According to many of the respondents, their kratom use was meant to address symptoms including pain, low energy, depressed or anxious mood. Additionally, a large proportion, if not the majority, of use was intended as a means to reduce or abstain from prescription or over the counter drugs to treat ailments for which kratom’s side effect profile was more tolerable (e.g. sedation and withdrawal in opioids) (Garcia-Romeu et al. 2017 (unpublished survey); Grundmann 2017; Henningfield et al. 2017; Pain News Network 2017). At the time of this writing, kratom products are lawfully marketed, though FDA and marketers are in continuing discussions concerning whether kratom products are subject to new dietary ingredient notifications requirements or should be treated as “old dietary ingredients,” and what the most appropriate regulatory framework is, though these are beyond the scope of this review (Botanical Education Alliance 2017; Myers and Long 2016; Pocan et al. 2016).

For decades in SE Asia, and increasingly in the US, kratom has been used as a substitute for opioids for relief of pain, opioid withdrawal, and maintenance of abstinence from prototypic dependence-producing opioids. Hassan et al. (2013) attributes most kratom consumption in SE Asia as at least initially motivated by what they and others term “instrumental” or “instrumentalized” consumption as a means of achieving work goals and not withdrawing from such obligations. In contrast to daily opioid use, such kratom consumption is more likely associated with beneficial occupational and social outcomes (see also Assanangkornchai et al. 2007; Swogger et al. 2015; Grundmann 2017; Pinney Associates 2016, Attachment 1; Singh et al. 2014; Vicknasingam et al. 2010). Similarly, the scientific and ethnographic literature often describes kratom consumption as primarily motivated by the plant’s “useful,” “beneficial,” “labor sustaining,” “therapeutic,” “mood,” and “well-being” enhancing, and “instrumental” attributes. Additionally, some people list kratom use meant to act as a substitute for drugs to which they had become dependent. (Aziz 2014; Hassan et al. 2013; Ward et al. 2011; Warner et al. 2016).

There are parallels in motivations for consumption of coffee, tea, and other caffeinated beverages which are often reported as being used for its alerting effects, sustaining performance and enhancing mood (Addicott 2014; Borota et al. 2014; Cappelletti et al. 2015; Goldstein et al. 2010; Meredith et al. 2013). As is the case with caffeinated beverages, high-dose chronic kratom consumption, as appears more common in SE Asia than the US, can lead to signs consistent with dependence with difficulty abstaining, as well as apparent withdrawal symptoms (e.g., Aziz 2014; Goldstein et al. 2010; Hassan et al. 2013; Meredith et al. 2013; Ward et al. 2011; Warner et al. 2016). The intertwining of these factors, for caffeinated product consumption as for kratom product consumption, requires close attention to detail in distinguishing “dependence” without harm from negative social effects from “addiction.” Moreover, as is the case with respect to caffeinated beverages, common levels of kratom consumption are not generally associated with adverse health effects. Another parallel with caffeinated beverages distinguishes caffeine and kratom from substances such as cocaine which in some cultures have been used and can be used with relatively low risk of adverse effects when consumed orally (Biondich and Avner 2016; Llosa 2007). Even with a substance as potentially toxic and addictive as cocaine, the oral route of consumption provides slow onset kinetics and low dosing that carries an overall far safer profile than cocaine by its more typical modern routes of administration by smoking, nasal insufflation, and injection which cocaine lends itself to, but kratom and its mitragynines do not as discussed in Factor 3 (Karila et al. 2008; Llosa 1994) Thus, consumption of kratom, like caffeine, is typically by the oral route and not “in the face of harm or personal or recurrent social problems” as is a common defining feature of dependence disorders (i.e., “addictive” or “substance use” disorders) as discussed further in this review. These factors taken together contribute to the rationale that the APA has used to refrain from formally recognizing caffeine substance use disorder in the DSM-5 (as in earlier editions) even though it includes criteria for diagnosis of caffeine intoxication and withdrawal disorders (APA 2013).

Similarly, whereas kratom can cause signs consistent with dependence without typically causing harm or social problems (Aziz 2014; Hassan et al. 2013; Henningfield 2015; Lanier et al. 2016; Ward et al. 2011; Warner et al. 2016), and that “it also carries some abuse liability” at very high doses (as concluded by Ward et al. 2011, p. 1001), at least in the US, consumption is generally considered volitional and for general and/or specific desired effects.

The apparent worst-case scenario conditions for very heavy consumption appears to have been in SE Asia among laborers who were required to work at heavy labor tasks for long hours and often in extremely high heat conditions. Kratom leaves were plentiful and heavily relied upon to enable such work. This was described in *The Chemist and the Druggist* (1930) in a short article titled “Kratom Eaters” that described the effects of chewing 10–30 kratom leaves three times per day by laborers to prolong their ability to tolerate “arduous work”, extreme fatigue and “torrid heat…” and that “the habit is stated not to be harmful since the kratom eater does not change his character.” Note that this quantity of kratom appears extremely high compared to typical kratom consumption; however, the article included no estimate of alkaloid content or nature of the leaves that would permit quantitative comparison to today’s products and practices. Singh et al. (2014), in a review of dependence and potential withdrawal, also described very heavy consumption among workers but also by many people as a social enhancer with family and friends, with about 79% of such consumers using daily. They stated that “Most users share the belief that it is better to consum[e] Kratom in order to improve work performance than using illicit stimulant-drugs which could also be more expensive.” It was observed that in contrast to the view of opioid users, kratom users were seen as “diligent” and “hardworking” despite the view that they were dependent and many experienced mild symptoms consistent with withdrawal upon termination of use. Similarly, Suwanlert (1975) described “regular kratom users” as having an increased tolerance for work and “increased calm” (Aziz 2014).

### Animal data on drug discrimination

There have been no studies of the discriminative stimulus effects of kratom as an herbal product. A preliminary study by Harun et al. (2015) investigated the discriminative stimulus effects of MG in rats. Rats acquired the MG discrimination (15.0 mg/kg, i.p.); however, approximately 100 trials of training (50 MG and 50 vehicle sessions) were required before the rats could discriminate MG from vehicle. Note this is longer than typically required suggesting the possibility that the discriminative effects were not as distinct as for many opioids and stimulants to which discrimination training can be accomplished within 20–45 test sessions (Swedberg and Giarola 2015; Young 2009) though there is wide variability in the amount of required training across studies (Solinas et al. 2006). MG substituted for the morphine discriminative stimulus in a dose-dependent manner, suggesting pharmacological similarities between the two drugs. Generalization to morphine occurred at the MG dose of 15.0 mg/kg, i.p., and was not seen at the 1 or 3 mg/kg, i.p. dose. Based on the estimated bioavailability of orally administered MG of 3.03% (Parthasarathy et al. 2010), this 15 mg/kg i.p. dose would equate to an oral dose of about 495 mg/kg in rats to produce an effect that resembles 5 mg of injected morphine. Converting this to a human equivalent dose based on body surface area, suggests that extremely high dosage (e.g., more than 5 g of MG taken orally) would be required to produce equivalent effects in humans. Additional research characterizing the discriminative effects of kratom’s alkaloids, singly, in various combinations, and possibly in the form of typical kratom leaf extracts, would be useful. Nonetheless, the fact that MG could substitute for morphine supports its consideration for placement in the CSA, as well as human self-reported use as an alternative to or substitute for opioids (Garcia-Romeu et al. 2017 (unpublished survey); Grundmann 2017; Henningfield et al. 2017; Pain News Network 2017).

#### Rewarding effects

There have been no reported laboratory studies of self-administration of kratom extracts or MG in animals and no human abuse potential studies. Clinical reports and testimonials suggest that in contrast to the effects of increasing the dose of substances of high abuse potential (e.g., prototypic opioids, stimulants, and sedatives), increasing the dose of kratom is more likely to produce undesirable gastrointestinal effects, constipation, lethargy, and little additional mood enhancement (Henningfield 2015; Pinney Associates 2016; Attachment; Raffa 2015; Ward et al. 2011; Warner et al. 2016). A conditioned place preference (CPP) evaluation of intraperitoneally administered kratom extract and MG in rats suggested weak, but significant, rewarding effects at extremely high dosages relative to what humans can apparently tolerate and normally take (Sufka et al. 2014; Pinney Associates 2016).

#### Relevance of doses to humans and equivalencies with other unscheduled drugs

Reports of kratom consumption show that kratom is virtually exclusively consumed via the oral route. Although it is possible to extract MG in laboratories as is done for research and to then inject the substance, as is the case with caffeine (Rush et al. 1995), this has not been reported in the US or Southeast Asia where it is relatively easy to find parentally administrable forms of typical stimulants and opioids of abuse provided by illicit drug manufacturers. Oral absorption of MG is slow, prolonged, and was incomplete, with a calculated absolute oral bioavailability value of 3.03% (Parthasarathy et al. 2010). On the other hand, in the US and SE Asia, the vast majority of kratom users appear satisfied to ingest leaf material in the form of extracts, beverages, and powdered leaf added to foods or swallowed as capsules. The concentrations of MG, 7OH MG, and other alkaloids vary widely across kratom strains and probably as a function of weather, time of harvesting and other factors, as is the case with caffeine and other naturally occurring alkaloids in botanicals (Fox et al. 2013; Kratom News 2017; Sridevi and Giridhar 2014).

Poppy seeds and hemp provide relevant perspectives on the importance of dose and bioavailability as neither are commonly abused or treated as substances of abuse though both contain controlled substances. Poppy seeds are harvested from the opium poppy and contain a mixture of opium alkaloids (e.g., morphine, codeine, thebaine). Depending upon the seeds, harvesting practices, and processing, the content of opium alkaloids in the poppy seeds on, for example, a poppy seed bagel may range from a few micrograms to a few milligrams of opium alkaloids, whereas strudel, with layers of seeds may contain higher levels. Consumption of such foods can yield positive opioid test scores in workplace drug testing (Lachenmeier et al. 2010; Moeller et al. 2004; Thevis et al. 2003; U.S. Anti-Doping Agency 2014). Similarly, consumption of hemp seeds and oil, products not normally consumed, can also produce positive drug test (Fortner et al. 1997; Leson et al. 2001); however, the DEA (2003) exempts “THC-containing industrial products, processed plant materials used to make such products, and animal feed mixtures, provided they are not used, or intended for use, for human consumption….”

### Factor 2: scientific evidence of its pharmacological effect, if known

The pharmacology of kratom, and its mitragynines have received increasing study in recent decades, particularly in laboratories in Japan and SE Asia (e.g., Warner et al. 2016). The following discussion draws from original reports, Warner et al. 2016, and the 2015 testimony by Henningfield.

Over 40 different constituents have been isolated from kratom (EMCDDA 2015; Gogineni et al. 2015), and kratom leaves have been found to contain over 25 alkaloids (Tanguay 2011; Hassan et al. 2013). The alkaloids MG and 7-OH-MG are believed to be the primary active alkaloids in the plant (Tanguay 2011; Warner et al. 2016). The total alkaloid content in kratom leaves ranges from 0.5 to 1.5% (Hassan et al. 2013). MG makes up approximately 60% of this extract with 7-OH-MG accounting for only up to 2% (Prozialeck et al. 2012; Philipp et al. 2010; Kapp et al. 2011). In a recent study by Kruegel et al. (2016), the authors found only trace quantities of 7-OH-MG (by mass spectrometry) in their extractions of the raw plant material, and concluded that it is doubtful that 7-OH-MG is a universal constituent of all Mitragyna speciosa preparations and is unlikely to generally account for the psychoactive properties of this plant.

MG is an indole-containing alkaloid, structurally similar to yohimbine, a component of the common dietary supplement yohimbe (Hassan et al. 2013; Prozialeck et al. 2012; Rosenbaum et al. 2012). Both MG and 7-OH-MG exhibit nanomolar binding affinities for the μ-opioid receptors and possess functional activity in tissue assays (Takayama et al. 2002; Matsumoto et al. 2004). In addition, the antinociceptive effects of MG and 7-OH-MG in several rodent models are also inhibited by naloxone (Matsumoto et al. 1996; Matsumoto et al. 2004; Takayama et al. 2002). However, MG was found to produce markedly less respiratory depression than codeine (Macko et al. 1972).

MG produces diverse effects that suggest actions as a partial mu-opioid agonist (e.g., Kruegel et al. 2016; Matsumoto et al. 2004; Prozialeck et al. 2012) as well as adrenergic and serotonergic mediated effects (e.g., Boyer et al. 2008; Hazim et al. 2014; Idayu et al. 2011). More recent research is beginning to elucidate the neuropharmacological mechanisms by which MG can produce potential desirable therapeutic effects such as relief of pain with low undesired effects such as respiratory depression and abuse potential. Its non-morphinan derived and non-opioid resembling molecular structural scaffold functions at least as a partial agonist at the μ-opioid receptor with a signaling bias for G-protein-mediated pathways without recruitment of beta-arrestin2, suggesting that it is among the substances, referred to as receptor-biased agonists, that represent a new category of safer analgesics (Kruegel and Grundmann 2017; Kruegel et al. 2016; Váradi et al. 2016).

In animal models, MG exhibits activity on supraspinal μ- and δ-opioid receptors causing its characteristic analgesic effects (Rosenbaum et al. 2012; Hassan et al. 2013; EMCDDA 2015; Prozialeck 2012). Studies of interactions at the cellular level suggest that neurotransmitter released from the nerve endings at the vas deferens is inhibited (Prozialeck et al. 2012). This inhibition is suggested to occur through the obstruction of neuronal calcium (Ca2+) channels (Hassan et al. 2013; Philipp et al. 2010). Blocked stimulation of serotonergic 5-HT2A receptors and stimulation of postsynaptic alpha-2 adrenergic receptors are thought to contribute to stimulant activity (Rosenbaum et al. 2012; EMCDDA 2015).

The CNS effects of the mitragynines appears related to the binding affinities exceeding that of morphine at the δ- and κ- opioid central receptors (Prozialeck et al. 2012). Moreover, 7-OH-MG provides high opioid receptor affinity with agonist properties (EMCDDA 2015; Prozialeck et al. 2012). While polarity is increased due to the additional hydroxyl group on 7-OH-MG as compared to mitragynine, increased activity of 7-OH-MG is otherwise not well understood (Prozialeck et al. 2012).

Unlike the preclinical models, Kruegel et al. (2016) showed that MG acted as a partial agonist at human μ-opioid receptors (EC50 = 339 ± 178 nM; maximal efficacy (Emax) = 34%), and that it did not result in recruitment of beta-arrestin2 providing a potential mechanism for low abuse and respiratory depression liability. In contrast, at human κ-opioid receptors, MG was a competitive antagonist (IC50 = 8.5 ± 7.6 μM; pA2 = 1.4 ± 0.40 μM), fully inhibiting the activity of the reference agonist U-50,488. Similarly, MG acted as an antagonist at human δ-opioid receptors, but with very low potency. The other major natural alkaloids paynantheine, speciogynine, and speciociliatine showed no measurable agonist activity at any of the human opioid receptors at concentrations up to 100 μM, and only weak antagonist effects were observed. This partial agonist activity is consistent with the observation that MG was found to produce markedly less respiratory depression than codeine (Macko et al. 1972), as well as the lack of respiratory depression-related deaths discussed elsewhere in this report.

An alkaloid that occurs in very low concentrations in most leaf material and is apparently very low in many marketed products, 7-OH-MG was also characterized and found to be a potent partial agonist at human μ-opioid receptors (EC50 = 34.5 ± 4.5 nM; Emax = 47%). Further, it acted as a competitive antagonist at both human κ-opioid receptors (IC50 = 7.9 ± 3.7 μM; pA2 = 490 ± 131 nM) and human δ-opioid receptors (IC50 > 10 μM). The partial agonist activity of MG and 7-OH-MG at the human receptors was further confirmed in antagonist experiments, as both compounds were able to partially inhibit the response elicited by the full agonist [D-Ala2, N-Me-Phe4, Gly-ol5]-enkephalin (DAMGO). As with MG, the partial agonist activity of 7-OH-MG is consistent with the lack of respiratory depression-related deaths discussed elsewhere in this report.

Although MG (Warner et al. 2016; Hassan et al. 2013) and sometimes 7-OH-MG (Matsumoto et al. 2006) have been reported as 13 times more potent than morphine, many of the references used to support this figure appear to not actually report this finding. In fact, a wide range of findings have been reported for MG and 7-OH-MG related to relative potency (the amount needed to produce an effect) and strength (the maximal effect, e.g. guinea pig ileum muscle twitch, or analgesic effect on mice placed on an uncomfortable but not burning hot plate). These are often reported in article abstracts and in turn by the media as “morphine” or “opioid” like effects with little context for what was actually studied, what was found, and its relevance or lack thereof to human use and effects. More research is clearly needed to more clearly elucidate the receptor binding profiles and the diverse and probably complex mechanisms of action of the alkaloids of kratom singly, in combination, and as commonly occurs in marketed products and brewed extracts.

### Factor 3: the state of current scientific knowledge regarding the drug or other substance

Kratom leaves and crushed or powdered leaves are readily available on the internet and in stores in most states, but this material is not reported to be used by nasal insufflation, smoking, or intravenously (in contrast to opioids and stimulants that are commonly used by such diverse routes to speed absorption and intensify their effects). In addition to the apparently low potential to produce strong effects sought by persons who abuse drugs, the physical nature of the leaf material with its high ratio of cellulose fiber likely deters use by such routes because large amounts of material would need to be placed in the nose or smoked to produce effects. By analogy, caffeinated products are also not reportedly used by injection, smoking, or snorting likely in part because although some cocaine-like effects are possible when caffeine is given by injection, it is not a comparable euphoriant and is preferred by the oral route (Rush et al. 1995; Garrett and Griffiths 2001).

In SE Asia where the raw material is plentiful and there are many clandestine laboratories for synthesizing substances for abuse, kratom-derived products have not been a target, likely due to the apparently low abuse liability of MG and limited euphoriant-like effects produced by the product. Furthermore, in contrast to the effects of increasing the dose of substances of high abuse potential (e.g., prototypic opioids, stimulants and sedatives), increasing the dose of kratom is more likely to produce undesirable gastrointestinal effects, constipation, lethargy, and little additional mood enhancement.

The pharmacodynamic effects of kratom have been summarized in reviews (see Prozialeck et al. 2012; Warner et al. 2016). Kratom users can expect to experience full effects in about 30–60 min after ingestion, although onset can be noticeable within about 10–20 min. The half-lives of MG and 7-OH-MG are about 3.5 and 2.5 h, respectively. Both are eliminated from the body primarily with the urine (Neerman et al. 2013; Prozialeck et al. 2012). The pharmacokinetics following oral administration of MG in humans has been proposed as a two-compartment model based on the observed kinetics in ten healthy human male volunteers (Trakulsrichai et al. 2015). Prior food consumption or taking kratom in capsule form can delay the initial response. The effects of kratom typically last about 5–7 h, with the strongest effects at about 2–4 h after ingestion, although weak aftereffects can be felt as late as the next day (Rosenbaum et al. 2012; Prozialeck et al. 2012; Scott et al. 2014; Maruyama et al. 2009). Current pharmacokinetic data in both animals and humans is limited, and there appears to be a significant variability within each species and differences between species in terms of MG pharmacokinetics.

Approximately 1–5 g of raw leaves, which is defined as a low to moderate dose, will yield mild stimulant effects (EMCDDA 2015; Prozialeck et al. 2012). The onset of effects begins about 10 min or more after using a few grams of dried leaves (EMCDDA 2015). This dosage is often related to the stimulant effects commonly used by labor workers in SE Asia to fight fatigue (Prozialeck et al. 2012), and potentially increase alertness, sociability, and sexual desire (EMCDDA 2015). At this dose, the user may also possess normal to slightly constricted pupils and blushing. Unwanted side effects are generally minimal; however, anxiety and internal agitation have been described (Prozialeck et al. 2012). When exceeding 15 g of kratom leaves, one would expect to experience stupor, mimicking the effects associated with opioids (EMCDDA 2015; Prozialeck et al. 2012). Initially, sweating, dizziness, nausea, and dysphoria will often result. These effects quickly subside and are followed by calmness and a dreamlike state (EMCDDA 2015).

These findings are relevant to a consideration of abuse potential assessment in that the physiochemical properties of the substance in its available and used formulation influences the abuse potential of the substance (FDA 2017, page 4). In the case of kratom, which is marketed and used virtually exclusively in forms for oral ingestion, it seems reasonable to conclude that the physiochemical properties contribute to the relative safety and low abuse profile as compared to prototypic opioids and cocaine-like stimulants. This analysis is consistent with the conclusions of other recent reviews of the state of kratom science, which reveal a novel substance in need of further research to better understand its mechanisms of action, including the potential of various alkaloids singly and in various combinations, to produce tolerance and dependence, as well as effects, particularly in dosage forms relevant to oral consumption by humans.

### Factor 4: its history and current pattern of abuse

#### History

Whereas for new molecular entities, drug scheduling decisions are heavily based on their pharmacology and chemistry, evaluation of substances with extensive histories of use can also be guided by the patterns and consequences of use. The history of kratom’s presence and consumption in the US is recent compared to SE Asia and not well documented. Anecdotal reports, e.g., by Hmong immigrants in the 1980s and 1990, suggest that the Hmong and other immigrants from SE Asia likely brought kratom consumption practices to the US. However, there has been little documentation of this in the literature other than anecdotal reports (e.g., in Axelrod and Windell 2012, p.56). Broader commercial marketing of kratom products in the US by internet and in various health and natural food stores apparently began to increase in the early 2000s. A kratom industry survey estimated that by 2016, there were approximately 10,000 vendors selling kratom products in the US (Botanical Education Alliance 2016). Frequency of consumption of kratom, whether by consumption of home brewed liquids or commercial products, appears largely determined by individual preferences and reasons for consumption.

##### Typical mode of kratom consumption

The most common mode of consumption in the US is by liquids that are either prepared by consumers or purchased as manufactured products, often in 60 ml (2 oz) containers as have become increasingly popular for caffeinated energy based “shot” drinks and other supplements, although various surveys suggest that powdered leaf material which is added to foods and beverages or consumed in the form of capsules appears to be growing in popularity (Kratom Online 2017; Kratom Science 2013). Consumers who prepare their own liquids use both hot and cold-water extraction methods similar to making tea or coffee. Leaf material, as either whole leaf, or chopped or powdered (sometime sold in capsules), can be steeped or boiled, or cold water extracted. Lemon juice or other acids may be added to facilitate extraction. Sugar, honey, and other sweeteners and flavoring ingredients are often added to mask the generally perceived unpleasant and bitter taste of the liquids. A public health benefit of the general distasteful nature of the liquids might be to somewhat discourage accidental consumption by children, although bad flavor does not necessarily prevent toxic substance exposures and ingestion by children (e.g., Reed and Knaapila 2010; Mennella et al. 2013; Rozin et al. 1986).

In SE Asia, leaf chewing is common with ready access to trees or inexpensive harvested leaves. There have been some reports of leaf smoking in SE Asia but this does not appear common in SE Asia where product is readily available or in the US. This is in striking contrast to opioids which are rarely consumed as beverages or in foods and which are commonly used by smoking, injecting, and nasal insufflation (“snorting”), as well as by the oral route in SE Asia as well as the US and other countries.

#### US federal surveys

Among federal surveys, the youth and young adult targeted Monitoring the Future (MTF) survey and the Treatment Episode Data Set (TEDS) were evaluated. MTF data are available through 2016 and TEDS through 2014 (Monitoring the Future 2017; Substance Abuse and Mental Health Services Administration 2017). Neither of these surveys have reported kratom consumption or treatment seeking for kratom dependence, respectively. The National Survey on Drug Use and Health (NSDUH) is generally considered to be a sensitive indicator of emerging trends in substance abuse, including adoption of new substances, and it includes collection of self-reported new and novel products and substances by its open-ended questions. Thus, although it does not yet include kratom/MG-specific questions, since 2010 through the most recently published data release that covered 2014, there were a total of two (2) kratom mentions (unweighted—not nationally representative). By contrast, and over the same timeframe, mentions of oxycodone, heroin, cocaine, amphetamine, marijuana, and other prototypic substances of abuse were in the many thousands. Aspirin mentions ranged from 17 to 22 per year, while diphenhydramine mentions ranged from 12 to 29 per year.

The virtual absences of kratom in these surveys do not mean there has been no abuse or kratom dependence treatment seeking; however, it does reflect the absence of signals and the lack of recommendations from affiliate researchers and treatment clinics that kratom abuse or dependence treatment should be added to the surveys at this time.

#### Other federal data sources

##### Drug Abuse Warning Network (DAWN)

There have been no reports of kratom or mitragynines in the DAWN system; however, since DAWN monitoring ended as of December 31, 2011, all that can be concluded is that DAWN-detected signals were not occurring before 2012. It is telling, however, that when clearly high-risk products such as fentanyl emerged even in small geographic areas, DAWN quickly picked up associated problems. Kratom likely had at least a decade of widespread use without generating any reports in the DAWN system.

##### National Forensic Laboratory Information System (NFLIS)

The NFLIS is a monitoring system of the Diversion Control Division of the DEA that reports laboratory identifications of substances collected in law enforcement operations and cases nationwide (National Forensic Laboratory Information System 2016a, b). Nearly two million analyses of drugs and other substances are tested annually from more than one million distinct drug cases. These include findings on opioids, depressants and tranquilizers, hallucinogens, anabolic steroids, and stimulants. NFLIS reports are not measures of actual use, abuse, or effects. MG was first reported in the NFLIS system in 2010 (National Forensic Laboratory Information System 2016a). From 2013 to 2015, MG reports accounted for approximately 0.01% of total reports. Specifically, 181 MG reports were recorded in 2013 (out of 1,540,647 total reports), 137 in 2014 (out of 1,511,313 total reports), and 129 in 2015 (out of 1,549,466 total reports). In contrast, in 2015, the total number of reports for drugs of abuse were 395,767 (cannabis/THC); 272,823 (methamphetamine); 216,129 (cocaine); 187,868 (heroin); 45,584 (alprazolam); and 41,894 (oxycodone) (National Forensic Laboratory Information System 2016b).

As confirmed by NFLIS, kratom is available to persons who have been found with substances of abuse, yet kratom has not emerged as a substance of abuse by any of the federal surveillance systems. Nonetheless, as MG identifications were a new category, the DEA placed MG on its “watch list,” meaning essentially that laboratories and investigators are encouraged to be alert for products potentially containing MG and to be testing for MG.

### Factor 5: the scope, duration, and significance of abuse

As discussed in Factor 4 above, there appears to be little, if any, abuse of kratom in the US. To the extent to which benefits are provided by consumption of kratom-derived products, these appear possible with remarkably low risks of serious adverse effects as compared to opioids and there is little evidence or apparent risk that kratom products are used by routes other than oral beverage or food consumption, even though it is certainly theoretically possible to smoke, snort, or inject kratom extracts. Nonetheless, some people do consume kratom for purposes of getting “high,” “intoxicated,” or “wasted” as self-reported on various websites and the incidence as discussed in Section 2.5.1. The prevalence of such use among kratom users will be increasingly important to study.

#### Internet monitoring

There are many websites that focus specifically on drug misuse and abuse, some intended to discourage such use as well as those that appear dedicated to providing information in support of, if not to encourage, misuse and abuse of drugs. Many of the kratom-related postings involve what appear to be extremely high dosages of kratom substances and extracts, and self-made extracts from a variety of kratom sources. For example, users may combine several grams of kratom powder, several ounces of kratom leaves, and indeterminate forms of this or other substances. Some people have reported experiencing intoxication, euphoria, and other effects at these very high dosages, though typically their comparisons to other drugs provide a basis for understanding why kratom and kratom products apparently are rarely the substance of choice among people who seek abused drugs and are in search of better ways to get better highs and euphoria. There are self-reports of dependence and withdrawal, but these tended to involve extremely high intakes of kratom, apparently along with other substances.

Similarly, Swogger et al. (2015) conducted a qualitative analysis of first-hand descriptions of human kratom consumption that were submitted to, and published by, a psychoactive substance information website (Erowid.org) as “experience reports.” For such participation in the Erowid system people are asked to self-administer the substance, often at high dosage levels, and then report their effects over the next 8 h. A caveat, based on the experience of the authors of this report, is that Erowid site participants would seem more likely than people in the general population to be heavy users and poly drug users and abusers, including many in this survey with self-reported use and dependence to prototypic opioids, cocaine, and other drugs. Four general themes emerged as associated with kratom product consumption (see Swogger et al. 2015 for greater detail): (1) Positive experiences was the most prominent theme with euphoria occurring in 30.4% of the respondents especially at high dosages, relaxation in 23.6%, and increased energy in 8.7%. (2) Negative experiences including nausea, stomachache, and cramping occurred in 16.1%. This included alternating chills and sweats in 9.3%, dizziness and unsteadiness in 6.8%, and vomiting in 3.1%. (3) Neutral experiences occurred in about 10% of the respondents, which included numbness of the throat and mouth, visual alterations, and sedation. (4) Substitution occurred in 10.6%, meaning that 10.6% used kratom as a substitute for an unwanted substance. This included 9.9% who used kratom to relieve symptoms of withdrawal from another substance. Themes that emerged from these experience reports indicate that kratom may be useful for analgesia, mood elevation, and anxiety reduction, and may aid opioid withdrawal management. Negative response themes also emerged, indicating potential problems and unfavorable “side” effects, especially stomach upset and vomiting. 10.6% of individuals reported successfully using kratom as a substitute to help abstain from the use of other substances perceived as addictive and/or causing harm. These substances were primarily opioids, such as oxycodone and heroin, but also included benzodiazepines and antidepressants. In addition to substitution, 9.9% of the sample reported withdrawal symptoms after using kratom. The consumers generally perceived their withdrawal symptoms to be milder than, but similar to, those caused by withdrawal from opiates. Only 5% of the sample reported tolerance to kratom, including a willingness to take higher doses in order to achieve the same effect. Finally, 4.3% of experience reports referenced hangover-like symptoms such as headache and nausea on the day after ingestion of kratom.

#### Online anonymous surveys

Grundmann (2017) conducted an internet survey of 10,000 self-reported current kratom users, of which 8049 completed the survey and their results analyzed. Demographic findings included the following: primarily used by persons 31–50 years of age, 53% “married or partnered,” 89% “white,” 70% employed, 61% had insurance, 16% had Medicare or Medicaid, 14% “no insurance,” and 82% had “at least some college.” Fifty-seven percent had been using for 6 months to 5 years. They reported the following beneficial effects (respondents could report more than one beneficial effect): increased energy (79%), decreased pain (80%), increased focus (66%), less depressed mood (74%), less anxious mood (74%), reduced or stopped the use of opioid painkillers (46%%), reduced PTSD symptoms (16%), elevated mood (72%), other (15%). 99.35% answered “no” to the question asking if “medical or mental health care treatment needed because of kratom consumption?” 40.05% discussed their use with a healthcare provider. 20.93% reported negative effects that were “primarily gastrointestinal related including nausea and constipation.” The results are also generally consistent with testimonials reported to the AKA and which are summarized in the Pinney Associates 2016, attachment.

The Pain News Network (2017) conducted an online survey of over 6400 kratom consumers resulting in 6150 respondents for analysis. Treatment for chronic or acute pain was the most common reason for use (51.34%), followed by treatment for anxiety (14.15%), opioid addiction or dependence (9.24%), and depression (8.83%). For treatment of pain kratom was rated as “very effective” by 90.38%, and somewhat effective by 7.17%. 98.06 answered “no” to the question: “Do you think kratom is harmful or dangerous substance?” To the question, “Can you get “high” from using kratom?” 75.03% answered “no”, 22.58 answered “a little”, and 2.40 answered “yes”. In response to questions concerning what kratom consumers were likely to do if kratom is classified as a controlled substance and made illegal, 68.76% endorsed “use opioids to treat pain”, 66.19% endorsed “become more likely to be addicted and overdose on other substances”, and 51.55% “become more likely to consider suicide”. In response to the question “If kratom is made illegal, will you personally seek to buy kratom on the black market?” 17.10% endorsed “yes”, 43.26% endorsed “not sure”, and 29.63% endorsed “no”.

Two surveys, unpublished at the time of this writing, Garcia-Romeu et al. (2017) and Henningfield et al. (2017), both found similar results among its respondents.

Garcia-Romeu et al. found that among 2017 current kratom users, the primary reasons for use included alleviating pain (91% of respondents), anxiety (68%), or depression (65%). Additionally, 41% of respondents reported using kratom to reduce or eliminate prescription or illicit opioid use. Nineteen percent reported experiencing adverse effects resulting from kratom use, though these were mostly mild and were less than 24 h in duration.

Henningfield et al. (2017) conducted an anonymous, cross-sectional, online survey of 3000 current and former kratom users. While the analysis has not been fully completed at the time of this writing, top line survey results seem to support the conclusions that have been determined through the earlier surveys: that kratom is a viable method for some people to reduce or eliminate use of prescription and/or illicit opioids; that further laboratory research is required to determine the degree to which kratom can substitute for opioids; and that appropriate product labeling and regulation has the potential to mitigate the “opioid crisis,” instead of exacerbating it.

While these four surveys were convenience surveys, the convergence of findings across the four surveys suggest that together, they all add to our understanding of the effects of kratom and the motivations of its users. These results can guide regulatory authorities in determining the types of restrictions to access of kratom are justified based on the scientific evidence available; the benefits and risks of allowing continued access to the public; and the types of incentivizing claims and deterring warnings that are appropriate for these products.

#### Testimonials regarding benefits

Consistent with the surveys cited above, Attachment A to the 2016 Pinney Associates 8-FA provides testimonials from kratom users regarding the perceived benefits of kratom. The testimonials provide qualitative and personal insights that complement the quantitative and qualitative surveys by Dr. Grundmann and by the Pain News Network. The profile that emerges is that kratom is consumed primarily for therapeutic and quality of life enhancing reasons. For many, the reasons include its value as a natural remedy for ailments, pain and mood in particular, but with benefits including increased energy and focus. It is not possible to estimate what fraction of people with opioid substance use disorders are using kratom to reduce or abstain from opioids and this will be important to learn in efforts to address the problems of opioid abuse and overdose but these surveys confirm what has been known for decades in SE Asia: many opioid addicted people find kratom to be a path away from opioids, and a desirable path that help them restore productive occupational fulfillment and improve social and family relationships. These consumers believe that kratom is more satisfactory than conventional medicines with respect to apparent effectiveness and has fewer undesirable side-effects than conventional medicines. The relative absence of apparent abuse of kratom as measured by national surveys does not mean there is no abuse, but certainly the signal is very weak compared to many other substances that people seek help for in achieve abstinence.

### Factor 6: what, if any, risk there is to the public health

Kratom products have been widely marketed and consumed as dietary supplements and natural remedies since at least the early 2000s. In collaboration, the Centers for Disease Control and Prevention (CDC) and the FDA “estimated the number of emergency department visits for adverse events associated with dietary supplements in the United States using 10 years of data (from January 1, 2004, through December 3, 2013) from the 63 hospitals participating in the National Electronic Injury Surveillance System-Cooperative Adverse Drug Event Surveillance (NEISS-CADES) project conducted by the CDC, the FDA, and the Consumer Product Safety Commission” (Geller et al. 2015). They estimated an average of 23,005 such emergency department visits annually, with 2154 hospitalizations annually. One fifth of the supplement-related visits involved unsupervised ingestion by children. The vast majority of the emergency visits involved products used for weight loss, energy, and sexual enhancement involving substance such as kava, hydroxytryptophan, caffeine, ephedra, ginseng, and yohimbine root. Less than 2% involved products used for pain or arthritis relief and these included substances such as arnica, glucosamine and pokeweed. None were reported to have involved kratom or mitragynines. This does not mean that there actually had been none involving kratom or mitragynines, but certainly the public health signal through this major reporting system was very small and not indicative of a major public health problem.

In a recent review of the toxicology of MG and analogs, Ramanathan and Mansor (2015, p. 282) concluded as follows: “To date there have been no reports of fatal overdose of kratom *per se*. If there are such occurrences, they are probably the result of kratom products contaminated with synthetic adulterants.” This is consistent with other reviews of kratom pharmacology, toxicology, and epidemiology (Warner et al. 2016). In fact, if kratom products were banned from the market, it appears likely that many users would turn to the illicit market that would immediately expand to meet the demand. In that marketplace, there would be no oversight by FDA and no basis for consumers to be assured of product purity and contents. Moreover, the illicit market is also a competitive marketplace. It is reasonable to assume that many illicit product manufacturers and distributors would be likely to spike their products with various other substances in order to support their claims such as “Special Product X” (possibly with added synthetic cannabinoids to provide claimed increased mood altering effects); “Special Product Relief” (possibly with added synthetic opioids to increase pain relief); “Special Product S” (possibly with added synthetic stimulants to increase the stimulant effects) and so on. Replacement of the licit market (and a licit market that would hopefully thrive with increased FDA oversight) with the illicit market would invariably precipitate public health problems, serious adverse events, and associated overdose deaths that would pose far greater risks to the public health.

#### American Association of Poison Control Centers’ National Poison Data System (AAPCC-NPDS)

The AAPCC-NPDS is considered a timely and sensitive system for tracking the emergence of trends in use-related effects and for capturing relatively low frequency events. From 2000 to 2005, a total of two (2) kratom-related exposures were reported to AAPCC; however, from 2010 to 2015, a total of 660 kratom-related calls were received (increasing from 26 in 2010 to 263 in 2015) (Anwar et al. 2016). While the number of kratom calls for 2014 is not known, a reasonable proxy would be the 263 known kratom-related calls from 2015. In comparison, there were 55,151 diphenhydramine-related calls, 18,470 aspirin-related calls, and 1355 nicotine pharmaceutical-related calls in 2014 (e.g., nicotine gum).

#### Children and adolescent exposure related adverse events and deaths

The US Poison Control Centers received approximately 32 calls per day for exposures to opioids by children and adolescents from 2000 to 2015 (Allen et al. 2017). Most of these do not result in death, but between 2011 and 2015, there were 51 reported pediatric (children less than 6 years of age) deaths from “analgesics” as well as 11 from “antihistamines,” 15 from “cleaning substances,” 12 from "cold and cough preparations", and 13 from “batteries” among other substances (National Capital Poison Center 2017). Kratom products were not listed in these reports nor internet searches for local and national media that typically report such events as news stories. This does not mean that no such events have occurred, but it does suggest that the signal is very weak and that any exposures that have occurred have not been associated with severe consequences.

At least three factors plausibly contribute to the apparent low risk that kratom products pose to children, as well as to adults: (1) Low toxicity and harm potential of kratom and its alkaloids; (2) Poor taste of even commercially marketed products with a commonly described “yuck” factor that would be expected to discourage consumption by children; and, (3) Relatively low concentrations of alkaloids apparent in most marketed products and in raw leaf material. Nonetheless, with increased availability and use of kratom products, it would be expected that there will be increasing reports of accidental consumption by children. Minimizing such risks through appropriate packaging and labeling requirements is a potential benefit of regulation as a dietary ingredient and by other authorities of FDA.

#### Ex-US safety/toxicity data

Kratom (including it specific alkaloids) products are not listed in the 1961 and 1971 drug control conventions (Spillane and McAllister 2003), but several countries including Thailand and Australia have adopted control measures on kratom perhaps based on economic factors and/or misperceptions about its patterns of use and safety (Aziz 2015). For example, Thailand, which may have been the first to schedule kratom and whose action apparently influenced other countries did so primarily because kratom, being readily available in forests, could not be conveniently taxed and so controlling the substance may have been meant to reduce the substitution of kratom use for opioids and other medicines that were taxed (Cleversley 2013). Nonetheless, kratom consumption is quite high in SE Asia, with likely over one million regular adult users in Thailand alone. Heavy use by laborers, plentiful and inexpensive supplies of raw material, and hundreds of years of history of use have provided both experience in consumption as well as countless millions of exposures and opportunities for overdose deaths and other serious adverse health consequences if there were a high risk of such. Yet fewer than 100 serious adverse events associated with kratom consumption have been reported from SE Asia. A limitation of safety data from SE Asia is that much kratom consumption likely occurs in rural areas with limited reporting systems. Nonetheless, in contrast to opioid abuse and dependence, kratom consumption is not considered a major public health problem.

In an exploratory ethnographic survey of 149 long-term regular users of kratom (who chewed the leaves) in Thailand, the percentage of users reporting the negative effects of using kratom was relatively low (4–14% of all users) (Assanangkornchai et al. 2007). The negative effects included a perception of less productivity, a decreased sexual drive, fatigue, poor health, wasteful spending habits, dizziness, poor concentration and distractedness, difficulty sleeping, wasting working time, irritability, poor thinking ability, impaired memory, laziness, and social withdrawal. The perceived benefits of kratom consumption included helping users work longer and harder, feeling happy/sprightly, maintaining a good mood, sleeping soundly, and being healthy. In Thailand, the benefits of consumption, as reported by users and nonusers alike, appear to predominate over the negative effects, although these conclusions come from research that is more ethnographic characterization than clinical trial type research.

The safety of MG has also been evaluated in animal toxicology studies. Hassan et al. (2013) reviewed the data on the toxicology of MG as follows: In animal models, the toxicity of MG was claimed to be relatively low. Macko et al. (1972) found no evidence of toxicity, measured as tremors or convulsions, at doses as high as 920 mg/kg in dogs. A more recent study in rats reported lethal effects of 200 mg/kg total alkaloid extract of *M. speciosa* given intragastrically (Azizi et al. 2010). The actual amounts of MG as compared to other alkaloids were not reported by Azizi, and the specific relevance to human safety is limited, because humans consume far less than 200 mg/kg. Janchawee et al. (2007) reported lethal effects after an oral dose of 200 mg MG in rats.

Sabetghadam et al. (2013) administered MG (1, 10, 100 mg/kg, p.o.) to rats for 28 days. The groups of rats treated with the lower and intermediate doses showed no toxic effects during the study. Only relative liver weight increased after treatment with the high dose of MG (100 mg/kg) in both the male and female treatment groups of rats. Biochemical and hematological parameters were also altered, especially in the high dose treatment group, which corresponds to the histopathological changes. Another study, also mentioned below in a summary of addiction potential studies is relevant to safety although it was designed to assess physical dependence and withdrawal at very high dosage relative to typical human consumption (Yusoff et al. 2016). Laboratory rats were given 30 mg/kg/day i.p., equating to an oral dose of about 990 mg/kg – equivalent to over 800 human 2-oz doses. Some evidence of dependence and withdrawal were demonstrated but not lethality.

#### Deaths possibly involving kratom

To date, there have been no reports of fatal overdose that may be categorized as kratom-caused poisoning deaths, by criteria used by medical examiners and in emergency medicine reports although several deaths in the US may have involved kratom (Pinney Associates 2016; Raffa 2015). Although there has been little systematic study of the pharmacodynamic effects of kratom, there is little clinical or scientific evidence of respiratory depression, and this would be consistent with the absence of documented overdose deaths attributable to kratom. Fourteen deaths potentially related to kratom had been reported globally at the time of the DEA proposal to schedule kratom. Of these, nine occurred in Sweden and appeared to have been related to consumption of an herbal blend called Krypton that was adulterated with O-desmethyltramadol, an active metabolite of the analgesic drug tramadol, which has been documented to carry a risk of severe respiratory depression and overdose death (Backstrom et al. 2010). The other five—three in the US, one in Norway, and one in Thailand—included co-administration of other drug substances. As a result, the actual cause of death is not clear.

In its analysis, DEA reported the following regarding deaths potentially related to kratom in its Factor 6 analysis:

Deaths related to kratom exposure have been reported in the scientific literature beginning in 2009-2010, with a cluster of nine deaths in Sweden from use of the kratom product “Krypton’ (Kronstrand et al. 2011). Since then, five more deaths related to kratom exposure were reported in the scientific literature (Holler et al. 2011; Neerman et al. 2013; Karinen et al. 2014; McIntyre et al. 2015; Anwar et al. 2016), and at least 16 additional deaths connected to kratom exposure, have been confirmed by autopsy/medical examiner reports (mitragynine and/or 7-hydroxymitragynine were identified in biological samples). Of these deaths, 15 occurred between 2014 and 2016 (citing Autopsy/Medical Examiner (ME) reports on file with DEA).

DEA has not made available information that would enable assessment of the basis for concluding that kratom was “connected” to the “16 additional deaths” (beyond the 14 discussed above and elsewhere (Henningfield 2015; Warner et al. 2016)). As demonstrated by the referenced sources in the DEA document, there has never been a published report in the literature of a death solely attributable to kratom, but rather these reported cases involved the ingestion of kratom along with pharmaceuticals or controlled substances known to present risk of death. These reports as summarized by the FDA document seem to agree with the general finding that these deaths were not solely attributable to kratom and generally leave the potential role of kratom unclear (see also Wing 2017).

A recently published, peer-reviewed article outlines well the position of the published studies: “Although death has been attributed to kratom consumption, there is no solid evidence that kratom was the sole contributor to an individual’s death” (Warner et al. 2016). By any measure this is in stark contrast with what has been documented for most recognized drugs and substances of abuse. For example, CDC reported data for 2014, with limitations acknowledged, in which deaths were reasonably concluded to have been categorized substantially, if not exclusively, to an opioid (28,647 in 2014 or nearly 80 per day) or other drugs most notably including sedatives, alcohol, and stimulants (18,408 or about 50 per day) for a total of 47,055 or about 129 per day (Rudd et al. 2016). As mentioned earlier, the very low risk of overdose poisoning and serious adverse events does not mean that they have not and will not occur. However, given the two decades during which consumption has increased to an estimated two or more million consumers in the US, in addition to far more extensive consumption in SE Asia, this is a substance and category of product with a remarkable safety record.

### Factor 7: its psychic or physiological dependence liability

There have not been laboratory studies of physical or psychological dependence or abuse potential in humans caused by kratom. Suwanlert (1975) reported that following the chronic exposure to *M. speciosa* preparations, abrupt abstinence can be followed by opioid-like withdrawal symptoms in humans. Typical withdrawal symptoms include hostility, aggression, excessive tearing, inability to work, aching of muscle or bones, and jerky limb movements.

Regarding physical dependence, ethnographic studies in SE Asia, some testimonials appended to the Pinney Associates 8-factor analysis, and the surveys by Grundmann (2017) and the Pain News Network (2017) suggest that abrupt discontinuation may be accompanied by withdrawal symptoms that are qualitatively similar but generally weaker than those observed following discontinuation of opioids. However, such reports make it difficult to disentangle the emergence of preexisting symptoms that had been mitigated by kratom use from those that occur as a physiological rebound accompanying the abrupt discontinuation of kratom use in kratom-dependent people. More studies of kratom’s potential to produce physical dependence, tolerance, and withdrawal are needed to characterize the nature and severity, and determinants of abstinence-associated symptoms. It is not completely devoid of signs that could be consistent with abuse or dependence, but neither is there evidence of high abuse/dependence potential that would support the conclusion that CSA scheduling is indicated.

### Factor 8: whether the substance is an immediate precursor of a substance already controlled under this subchapter

Neither kratom nor any of the constituents in kratom or its alkaloids are controlled substances or are precursors of a controlled substance. They are not a morphinans by origin or molecular structure, and the science to date indicates that they differ significantly in from prototypic opioids of abuse with respect to abuse potential and safety.

## Conclusions and scheduling recommendation

We recommend further study of kratom and its alkaloids and further consideration of public health benefits and risks of its historical and current use before any regulatory actions are taken to restrict its availability. On the basis of the current state of the science and apparent public health impact, we do not recommend scheduling of kratom or any of its specific alkaloids under the CSA because it does not share the profile of prototypic morphine-like opioids with respect to abuse potential and safety, and surveys indicate that banning the products would put kratom users who are presently using kratom to abstain from opioids at risk of resuming opioid use and overdose. The surveys indicate that some people who consume kratom for other reasons may turn to illicit kratom sources which would not be regulated by the FDA, thus exposing them to the risks of the illicit market.

Kratom’s overall low potential for abuse and risk to public health and its extensive history of safe use, including findings from recent surveys in the US, suggest a strong benefit to risk profile that is within the range of current dietary supplements and many over-the-counter products However, if some extract of kratom, or single entity was used to develop a drug, that product and its active substance(s) would need to be thoroughly evaluated for abuse potential according to FDA’s 2017 Guidance and determined, consistent with the CSA, if the product should be placed in the CSA and if so, which schedule would be most appropriate.

The further research into kratom that is warranted would be impeded by restricting kratom by placement in the Schedule I of the CSA. Equally negatively consequential is that, Schedule I placement would effectively ban any lawful marketing of these products; and would deprive FDA of application of its regulatory mechanisms to inform and protect consumers through FDA-regulated labeling, packaging, product performance standards, oversite, and other actions FDA routinely implements in its regulation of foods, drugs, and dietary supplements.

History and patterns of use and effects confirm it benefits to consumers, however, it is important to acknowledged that such use and history does not meet criteria for approval of kratom as a drug for treatment of such disorders, nor has there been an application for approval of kratom or its constituents as new drugs. Rather, the natural products (i.e., powdered leaf) and manufactured extracts are marketed as dietary supplements. Regulation of kratom by the FDA could lead to the development of standards for product purity of natural leaf products and manufactured extracts, potential limitations on levels of active constituents in manufactured extracts, as well as product packaging and that would be in the interests of public health (Pinney Associates 2016).

An important consideration is that banning the availability of kratom through scheduling could precipitate public health problems that do not presently exist or are at very low levels, because this would shift the marketplace from a largely lawful retail market to illicit manufacturers and distributors with no regulated labeling, purity or content standards, or effective ability to remove adulterated products from the market. The four kratom user surveys conducted to date also suggest that some fraction of kratom users who were using kratom in order to reduce or eliminate opioid use would turn to illicitly marketed kratom and/or return to opioids, whether licit or illicit. That would be contrary to increasing efforts of public health organizations and the White House Opioid Commission (2017) to discourage opioid use and encourage the use of alternatives that carry less risk (Dowell et al. 2016).

The overarching public health and policy question is not “could kratom be regulated as a controlled substance” but rather “should kratom be so regulated.” From a pharmacological perspective, this review suggests, as concluded by Henningfield (2015) and Pinney Associates (2016) that a case could be made to place kratom in the CSA. In fact, if MG, for example, was a newly discovered active chemical entity in a medicine submitted for approval by FDA, and hence without decades of use in the community, it would certainly be evaluated for potential scheduling according to the CSA and FDA’s guidance (FDA 2017), and it might be recommended for scheduling following its approval as a therapeutic medicine. If that was the case, whether the appropriate schedule would be IV or V is not clear, but the fact that its overall potential for abuse and harm is well within the range of many nonprescription over-the-counter medicines and nonscheduled medicines, suggests that prescription requirements of new medicines would be considered appropriate and that sufficient experience might then lead to its switch from prescription to nonprescription status as often occurs with medicines with a satisfactorily documented safety profile.

## Going forward: research and regulation

The absence of an imminent threat to public safety does not imply that the status quo with respect to regulation is adequate to appropriately protect public health; nor does it imply that there is no need for further research. To the contrary, as suggested by the research summarized in this review, kratom-related research is at an early stage with many key gaps in knowledge. Similarly, regulation is vital to help ensure that lawfully purchased products are what they claim to be, are not adulterated, and are appropriately packaged and labeled. At the time of this writing, however, the threat of placement of kratom (specifically, MG and 7-OH-MG) into Schedule I of the CSA would serve as a major obstacle to research due to the barriers by the statutory requirements of such scheduling (Nutt et al. 2013; Scientific American Editors 2014).

While appropriate regulation is vital to kratom’s future, there is still much to be discovered about kratom’s potential benefits and harms and research, ideally including independent research supported by organizations such as the US National Institutes of Health would be important to serve public health interests as well as to advance the understanding of the both the molecule as well as its CNS mechanisms of action. This includes furthering recent findings suggesting that mitragynines may be functioning as biased opioid receptor agonists, thus helping to explain their apparent safety profile that sets them apart for prototypic morphine-like opioids of abuse.

### Acknowledgments of funding and grants

The time and effort of the authors on this article was supported by PinneyAssociates without input from any commercial interests. This report draws from an analysis of the eight factors of the US Controlled Substances Act that was supported by the American Kratom Association and was filed on the public docket of the Food and Drug Administration. The content is solely the responsibility of the authors and does not necessarily represent the official views of the American Kratom Association. The authors are grateful for the care and efforts of Evan Schnoll and Yolanda Green in the referencing and editing of the manuscript.

## Electronic supplementary material

ESM 1 (DOCX 24 kb)
