# Supplementary material for: The abuse potential of kratom according the 8 factors of the controlled substances act: implications for regulation and research
Source: Psychopharmacology (Berl). 2017 Dec 23;235(2):573–89. doi: 10.1007/s00213-017-4813-4 (PMC5813050; doi:10.1007/s00213-017-4813-4)
Supplement: Supplementary file 1 — (DOCX 24 kb) [file 213_2017_4813_MOESM1_ESM.docx]

**Supplemental Tables for Henningfield et al. December 1, 2017**

**Table 1. Temporary scheduling to avoid imminent hazards to public safety (U.S. Congress 2017)**

The CSA’s Subchapter I – Control and Enforcement, Part B - Authority to Control; Standards and Schedules, Section 811, (h) Temporary scheduling to avoid imminent hazards to public safety, includes the following provisions:

(1) If the Attorney General finds that the scheduling of a substance in schedule I on a temporary basis is necessary to avoid an imminent hazard to the public safety, he may, by order and without regard to the requirements of subsection (b) of this section relating to the Secretary of Health and Human Services, schedule such substance in schedule I if the substance is not listed in any other schedule in section 812 of this title or if no exemption or approval is in effect for the substance under section 505 of the Federal Food, Drug, and Cosmetic Act [21 U.S.C. 355]. Such an order may not be issued before the expiration of thirty days from—

(A) the date of the publication by the Attorney General of a notice in the Federal Register of the intention to issue such order and the grounds upon which such order is to be issued, and

(B) the date the Attorney General has transmitted the notice required by paragraph (4).

(2) The scheduling of a substance under this subsection shall expire at the end of 2 years from the date of the issuance of the order scheduling such substance, except that the Attorney General may, during the pendency of proceedings under subsection (a)(1) of this section with respect to the substance, extend the temporary scheduling for up to 1 year.

(3) When issuing an order under paragraph (1), the Attorney General shall be required to consider, with respect to the finding of an imminent hazard to the public safety, only those factors set forth in paragraphs (4), (5), and (6) of subsection (c) of this section, including actual abuse, diversion from legitimate channels, and clandestine importation, manufacture, or distribution.

(4) The Attorney General shall transmit notice of an order proposed to be issued under paragraph (1) to the Secretary of Health and Human Services. In issuing an order under paragraph (1), the Attorney General shall take into consideration any comments submitted by the Secretary in response to a notice transmitted pursuant to this paragraph.

**Table 2. Abuse potential related definitions as used by FDA (2017)**

**Drug abuse**: The intentional, non-therapeutic use of a drug product or substance, even once, to achieve a desired psychological or physiological effect. Therefore, abuse potential refers to the likelihood that abuse will occur with a particular drug product or substance with CNS activity. Desired psychological effects can include euphoria, hallucinations and other perceptual distortions, alterations in cognition, and changes in mood.

**Dependence – Physical [i.e., physiological]:** Refers to physical or psychological dependence. Physical dependence is a state that develops as a result of physiological adaptation in response to repeated drug use, manifested by withdrawal signs and symptoms after abrupt discontinuation or a significant dose reduction of a drug.

**Dependence - Psychological (or psychic):** Refers to a state in which individuals have impaired control over drug use based on the rewarding properties of the drug (ability to produce positive sensations that increase the likelihood of drug use) or the psychological distress produced in the absence of the drug.

**Tolerance**: A state that develops as a result of physiological adaptation characterized by a reduced response to a specific dose of drug after repeated administration of the drug (i.e., a higher dose of a drug is required to produce the same effect that was once obtained at a lower dose).

The presence of physical dependence or tolerance does not determine whether a drug has abuse potential. Many medications that are not associated with abuse, such as antidepressants, betablockers, and centrally acting antihypertensive drugs, can produce physical dependence and/or tolerance after chronic use.

However, if a drug has rewarding properties, the ability of that drug to induce physical dependence or tolerance may influence its overall abuse potential.

**Table 3. Summary of regulation of dietary ingredients under the 1994 Dietary Supplement Health and Education Act (DSHEA) (Abdel-Rahman et al. 2011; Commission on Dietary Supplement Labels 1997; Dickinson 2011; Larsen and Berry 2003; Swann 2016)**

Regulation of dietary supplements places an emphasis on safety and they are not subject to the New Drug Application (NDA) approval process required for medicines for the diagnosis, mitigation or treatment of a disease by affecting the structure or function of the body (i.e., “structure/function” claims).

- Dietary ingredients that were marketed as nutritional or dietary supplements in the United States prior to October 15, 1994 (the date of the Act’s enactment) do not require notifying FDA prior to marketing. Those ingredients that do not have adequate evidence of marketing prior to this date must be documented as a “new dietary ingredient” through a New Drug Ingredient notification (NDIN) to FDA. Manufacturers are responsible for determining if an ingredient was marketed before the referenced date (U.S. Food and Drug Administration 2016)
- The NDIN must include evidence supporting the conclusion that the supplement or ingredient will reasonably be expected to be safe. Upon receipt of an NDIN, the FDA must acknowledge its receipt within 75 days as well as notify the sponsor when the notification was received (filing date). After 75 days, the new ingredient or supplement may be marketed as long as FDA does not object or find inadequate the information contained within the notification.
- Health claims can imply the use of categories of foods and ingredients in maintaining good health. Somewhat more specific health claims can also be made but these must be determined to be “truthful and non-misleading” and subject to the Nutrition Labeling and Education Act of 1990 (NLEA), and the Food and Drug Administration Modernization Act of 1997 (FDAMA). Such claims are limited to claims of disease risk reduction, and may not be claims about the diagnosis, cure, mitigation, or treatment of the disease and must undergo FDA review prior to use.
